# Iatrogenic Botulism Following Botulinum Toxin Injection as an Adjunct to Abdominal Wall Reconstruction for Incisional Hernia

**DOI:** 10.7759/cureus.73773

**Published:** 2024-11-15

**Authors:** Joana Frazão, Rita Pera, Xavier De Sousa, Marta Fragoso, Paulo Mira

**Affiliations:** 1 General Surgery, Hospital Professor Doutor Fernando Fonseca, Amadora, PRT

**Keywords:** abdominal wall surgery, adjuvant techniques, botulinum toxin, complex incisonal hernia, iatrogenic botulism

## Abstract

Open ventral hernia repair is one of the most commonly performed surgeries by general surgeons worldwide. In the case of complex incisional hernias, there are adjunct techniques that can help abdominal wall reconstruction surgery, such as type A botulinum toxin (BTA), whose injection results in muscle relaxation and growth of muscle fiber length, allowing fascial closure without the need for advanced techniques.

We report a case of a male patient who underwent ultrasound-guided BTA injection in the abdominal wall and, five days later, was admitted to our emergency department with dysarthria, muscular weakness, dyspnea on small exertion, and constipation. There was a rapid worsening of respiratory failure, requiring invasive mechanical ventilation. After the exclusion of other neurological conditions, iatrogenic botulism was assumed to be the most likely cause.

There are adjunct tools that allow successful abdominal wall reconstruction, such as BTA, which is relatively safe. On rare occasions, systemic absorption of BTA can lead to systemic effects, such as iatrogenic botulism, that can be extremely severe. We have not found any case reports about iatrogenic botulism related to BTA use in abdominal wall musculature.

This is the first documented case of iatrogenic botulism related to BTA injection in the abdominal wall musculature. Given its severity, it is important that surgeons working with BTA are aware of these potential complications.

## Introduction

More than 20% of laparotomy patients develop an incisional hernia, so open ventral hernia repair is one of the most common procedures performed by general surgeons [1]. In high-risk patients, the rate of hernia formation is higher, so it is important to optimize patients and provide them with an effective repair during the first surgery [1]. In some incisional hernias, particularly in cases of loss of domain hernias or where the need for component separation is anticipated, there are adjunct techniques that can assist with abdominal wall reconstruction [1-4]. It is important for all patients to undergo a CT scan to better describe the hernia, plan the surgery, and predict if fascial closure is possible [1, 2]. The CT scan enables the calculation of several indices that help predict whether fascial closure can be achieved without the need for advanced techniques. The Carbonell Index states that direct defect closure with a Rives-Stoppa retromuscular repair would not be feasible without additional fascial release if the maximal defect width closely approximates or exceeds twice the rectus width [5]. While there are several definitions, loss of domain refers to a ventral hernia that is large enough that simple reduction and primary fascial closure either cannot be achieved without additional reconstructive techniques or carries a significant risk of complications due to raised intra-abdominal pressure [1, 3, 4, 6]. Tanaka’s Index is the ratio between the hernia sac volume and the abdominal cavity volume, providing a definition for a loss of domain hernia when it exceeds 25% [3, 5, 6].

Regarding adjunct techniques, the most common are progressive preoperative pneumoperitoneum (PPP) and BTA. Typically, PPP is used for patients with loss of domain hernias, while BTA is used for both hernias with loss of domain or with a width larger than 10 cm (W3 according to European Hernia Society Guidelines) [1-4]. BTA blocks acetylcholine release at neuromuscular junctions, thereby inhibiting action potential firing at neuromuscular synapses [1, 3, 4, 7]. When BTA is injected into the abdominal wall musculature, it results in the growth of muscle fiber length and its relaxation [1, 3]. This facilitates improved medialization of the fascia during abdominal wall closure and reduces intra-abdominal pressure [1, 3]. The effect of BTA usually begins two to five days after administration, peaks at four weeks, and lasts for six to nine months, so it is typically injected four weeks before hernia surgery [1, 3, 4]. Its injection is often guided by ultrasound to visualize the three lateral muscle layers to be infiltrated (external oblique, internal oblique, and transversus abdominis) [3, 4]. BTA is generally well tolerated by patients; however, some side effects have been described [3, 7-10]. The most frequent are self-limited local reactions at the injection site. Rarely, the toxin may diffuse around the injection site and reach the lymphatic circulation, leading to systemic adverse effects such as botulism [3, 7-10]. These adverse effects have mostly been described in cases of cosmetic use of BTA, especially those with repeated use [3, 7-10]. No cases of iatrogenic botulism have been reported when used in the abdominal wall musculature.

## Case presentation

**Figure 1:**
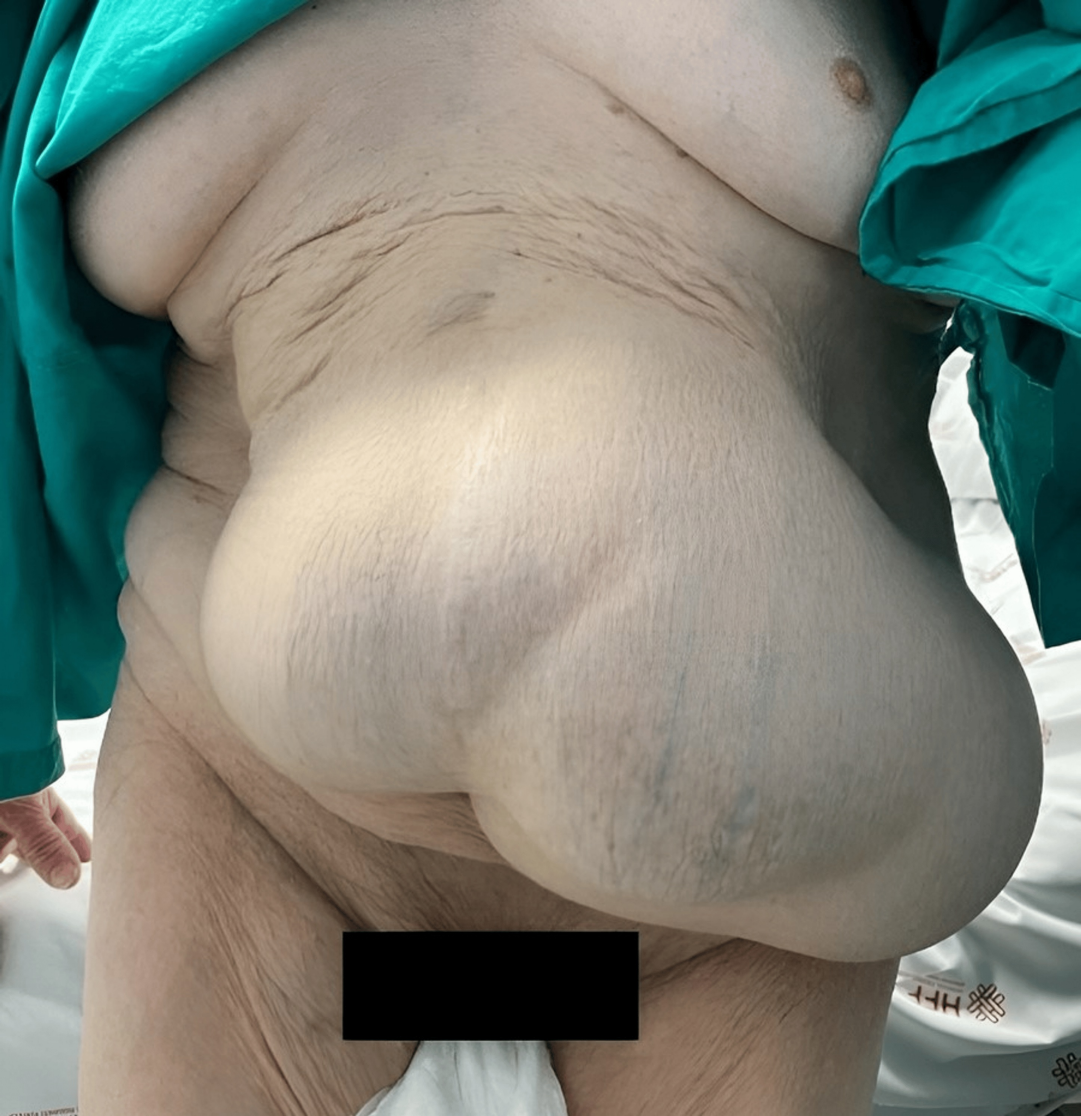
Incisional hernia classified as M2W3 (before PPP) PPP: progressive preoperative pneumoperitoneum.

**Figure 2:**
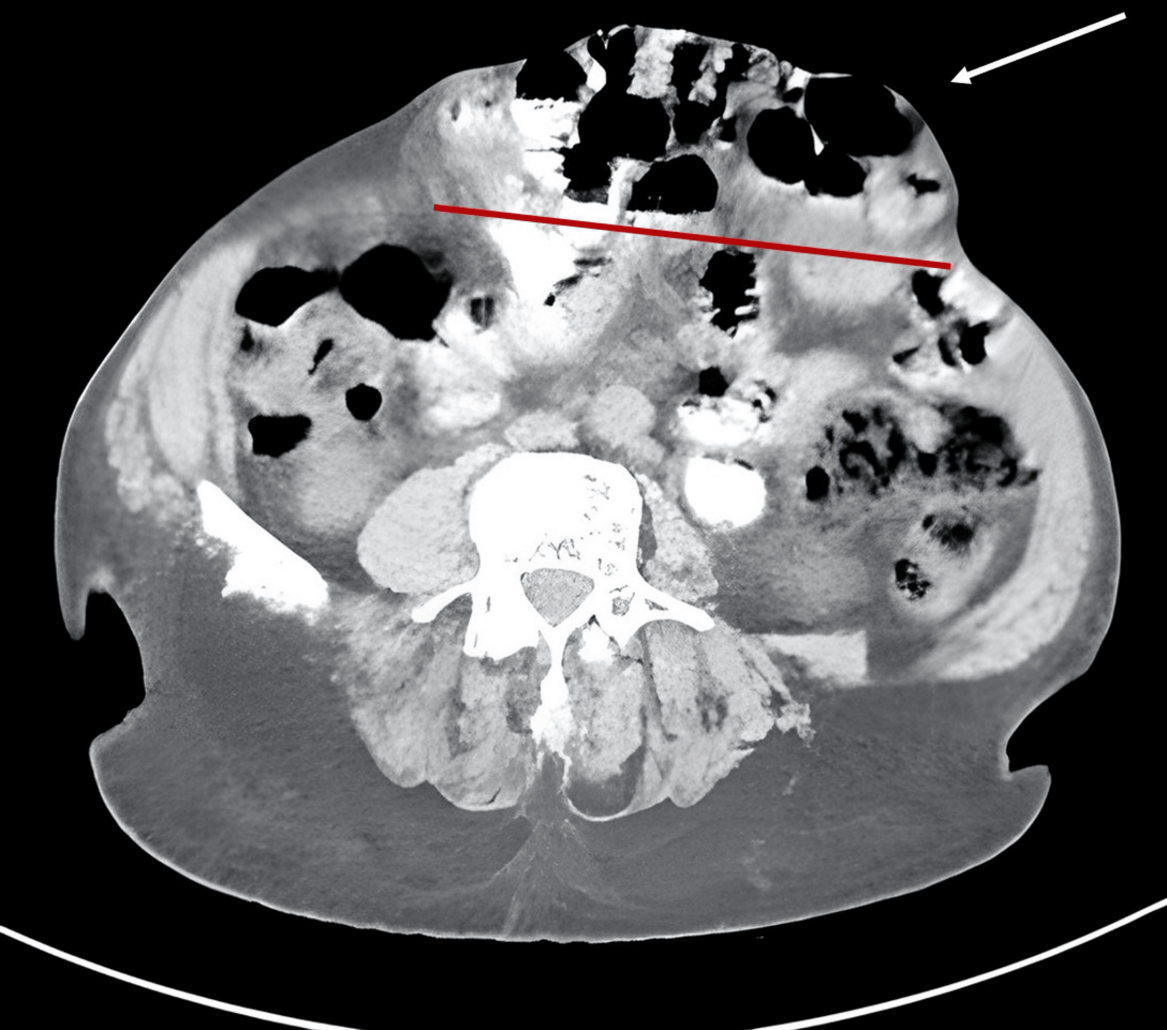
Abdominal CT scan showing the incisional hernia classified as M2W3

**Figure 3:**
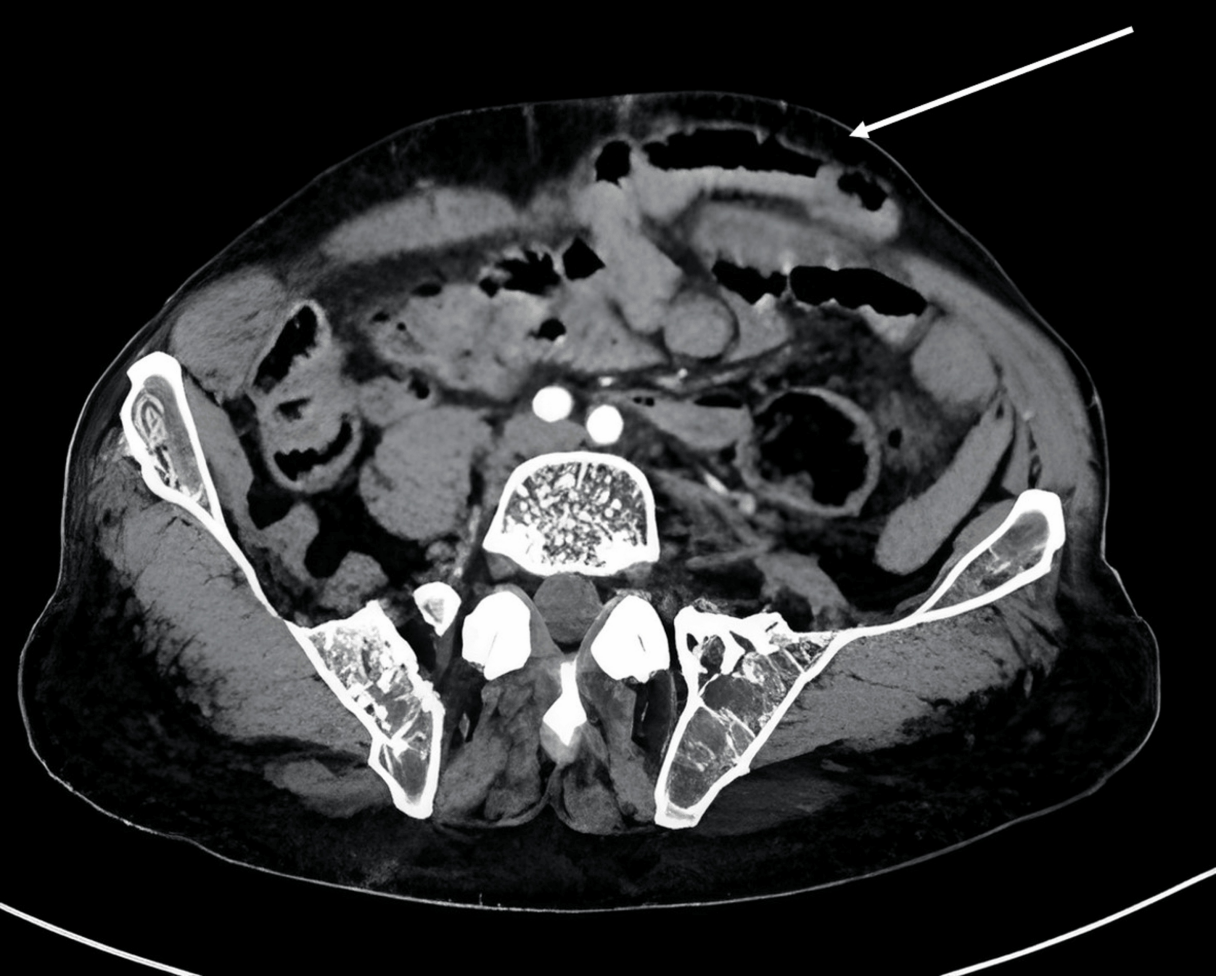
Abdominal CT scan showing the incisional hernia classified as M2W3

We report an independent 65-year-old male patient with a complex incisional hernia (Figure 1), classified as M2W3 according to the European Hernia Society Guidelines, which resulted from a past epigastric herniorrhaphy. Following the initial physical examination, a CT scan (Figures 2, 3) was performed, revealing an incisional hernia with loss of domain, as the Tanaka Index exceeded 25%.

Given the complexity of the case, BTA injection and PPP were proposed as adjunct techniques before the surgical correction. The patient subsequently underwent an echo-guided BTA injection (Dysport®, 500U, 3 to 5 injections per side, 50U per injection) as an outpatient procedure, without immediate complications.

Five days later, the patient presented to the Emergency Department with dysarthria, muscle weakness, dyspnea upon minimal exertion, and constipation. He was promptly admitted, and his respiratory function quickly worsened, necessitating invasive mechanical ventilation, leading to admission to the Intensive Care Unit (ICU).

After ruling out neurological disorders such as stroke, myasthenia gravis, and Guillain-Barré syndrome, iatrogenic botulism was presumed. The patient experienced several respiratory infections and required a tracheostomy.

Following progressive and significant improvement, the tracheostomy was closed, and the patient was discharged to a rehabilitation healthcare facility.

**Figure 4:**
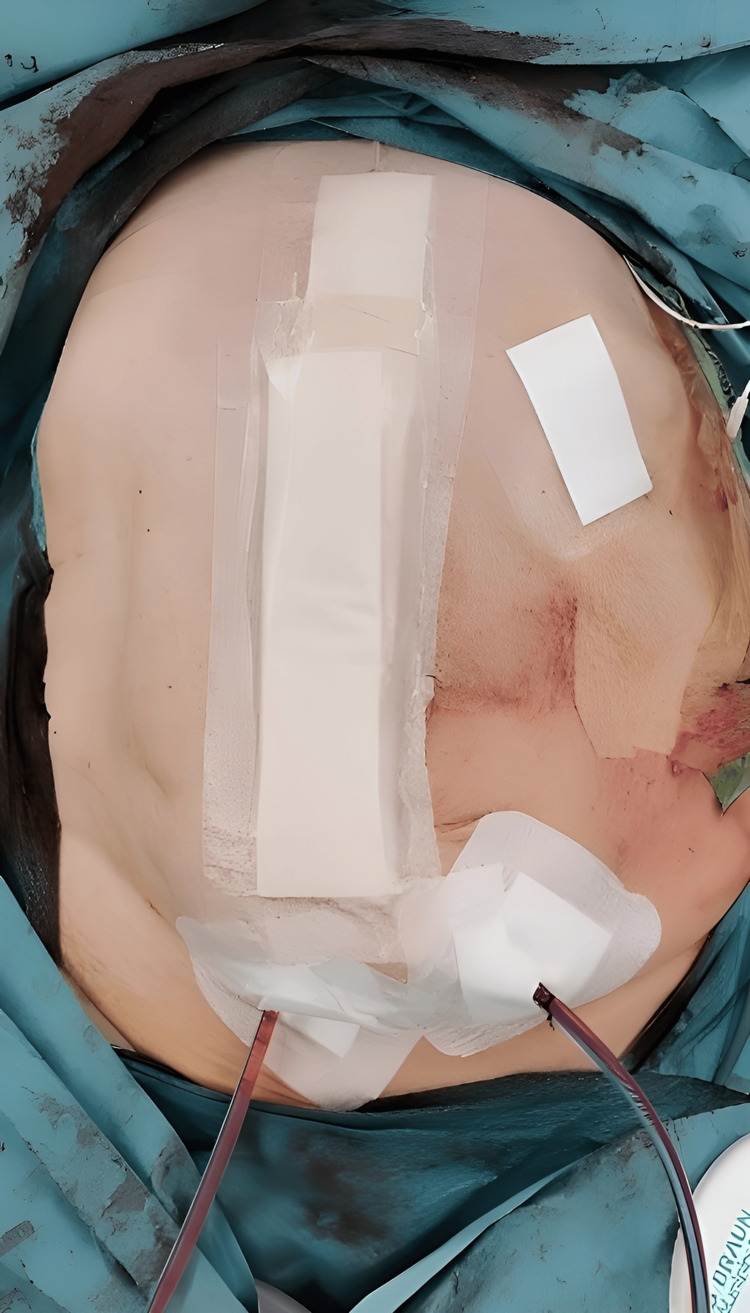
Immediate post-operative result

**Figure 5:**
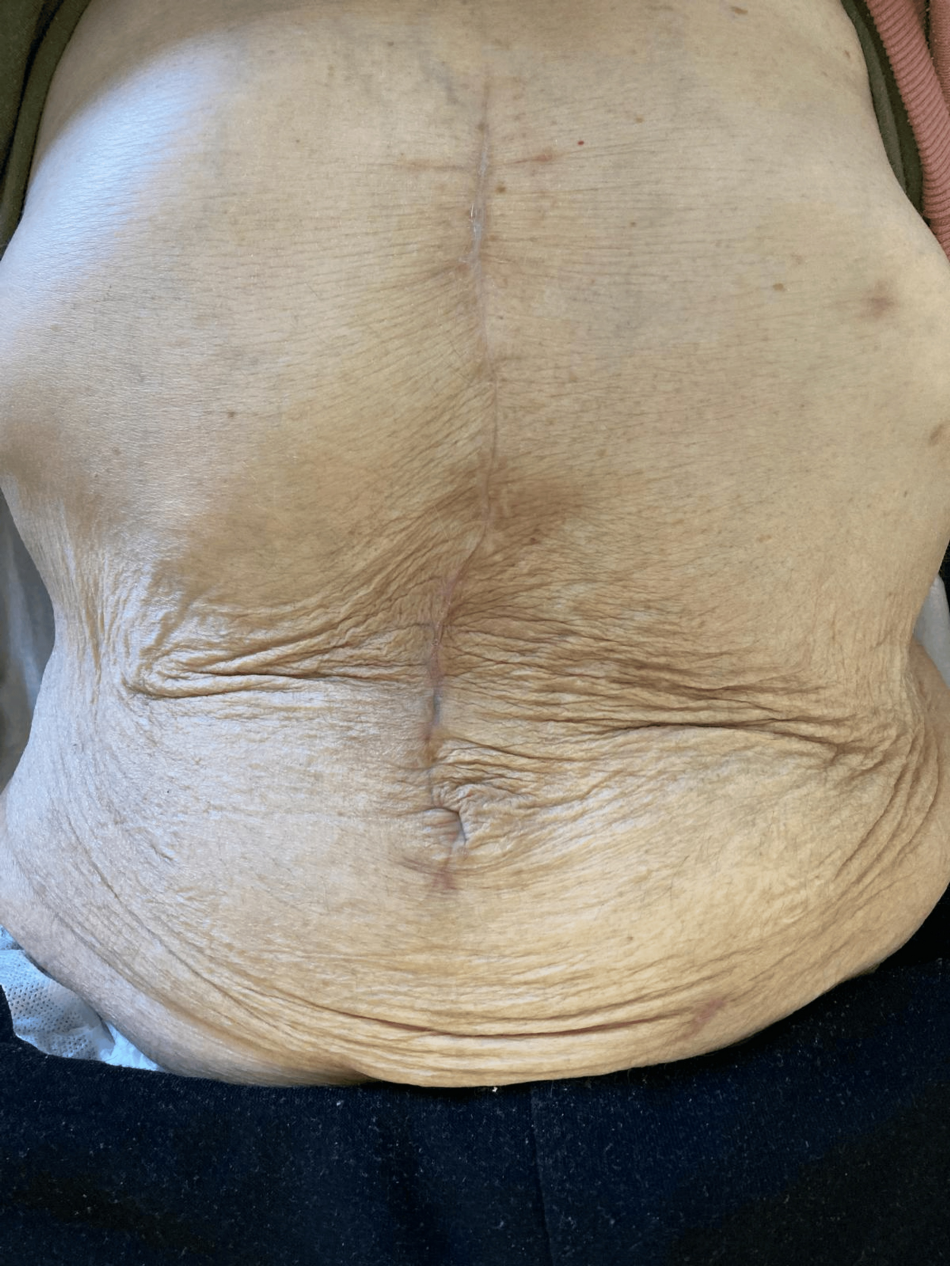
Final result: frontal view

**Figure 6:**
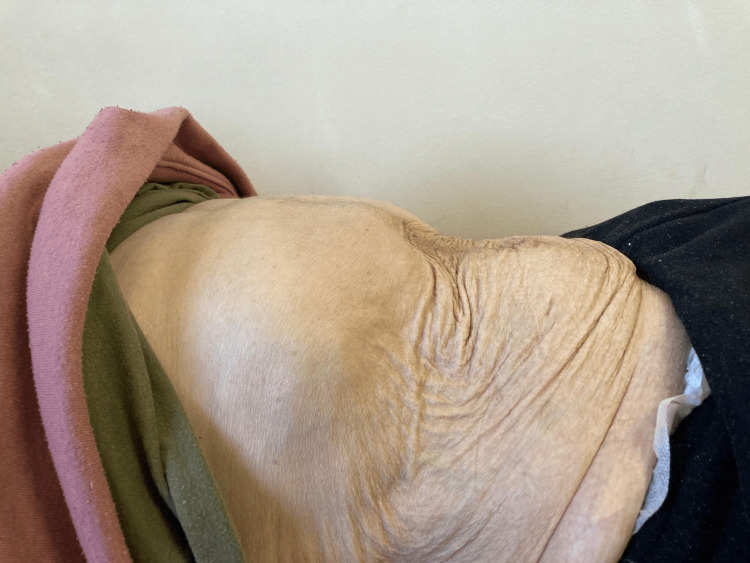
Final result: lateral view

One year later, he underwent PPP alone, and after two weeks, surgery was performed. A Rives-Stoppa hernioplasty was completed without the need for advanced techniques (Figure 4). There were no complications in the postoperative period. He continues with outpatient follow-up, with no recurrence and a marked improvement in his quality of life (Figures 5, 6).

## Discussion

There are several adjuvant tools used in abdominal wall reconstruction surgery that can sometimes eliminate the need to resort to advanced techniques. The most commonly used are PPP and BTA [1-4].

BTA is generally considered safe, although its widespread use and the constantly expanding indications raise safety concerns [9]. The therapeutic use of botulinum toxin dates back two centuries, when Kerner recognized its effect on skeletal muscles and parasympathetic function [8, 9]. It was first approved for the treatment of strabismus and blepharospasm in 1989, and scientific research into its use has continued to expand to this day [8, 9]. It was only in 2009 that Ibarra-Hurtado et al. reported the administration of BTA on the lateral abdominal wall muscles to induce transient flaccid paralysis and decrease the transverse diameter of the defect [11].

BTA is typically administered under ultrasound guidance, at 3 to 5 injection sites per side of the abdominal wall, approximately four weeks prior to surgery (when it reaches its peak of action) [1, 3]. Some adverse effects have been reported, most of which are mild and transient. Rarely, systemic absorption of BTA can occur, leading to iatrogenic botulism. Such adverse effects have been noted primarily in cases where BTA was used for cosmetic purposes [8, 9].

Iatrogenic botulism is a rare acquired neuromuscular junction disorder characterized by descending flaccid paralysis induced by botulinum neurotoxins as an adverse reaction to its use, which can be extremely severe [7]. The most common symptoms are weakness and dysphagia. Since this disorder is primarily diagnosed clinically, distinguishing it from neurological disorders with similar symptoms is critical [7]. There are no published case reports of iatrogenic botulism related to BTA use in the abdominal wall musculature.

In this case, the patient underwent BTA administration under ultrasound guidance without immediate adverse effects but developed symptoms of dysarthria, muscle weakness, dyspnea upon minimal exertion, and constipation five days later. Iatrogenic botulism was presumed, likely due to systemic absorption. After prolonged hospitalization with several complications, the patient underwent rehabilitation and showed progressive improvement. One year later, he underwent PPP without complications, and two weeks later, a Rives-Stoppa hernioplasty was performed without the need for advanced techniques. The patient recovered well.

## Conclusions

One of the most commonly performed surgeries by general surgeons worldwide is open ventral hernia repair. Complex incisional hernias may present a surgical challenge, emphasizing the potential value of adjuvant tools that enable correction without the need for advanced techniques, such as BTA. BTA works by blocking acetylcholine release at neuromuscular junctions, thereby inhibiting action potential firing at neuromuscular synapses and promoting muscle relaxation and growth in muscle fiber length. Most reported adverse effects are mild and localized, but in rare cases, systemic absorption can occur, leading to systemic symptoms, such as iatrogenic botulism, which can be extremely severe or even fatal. This is the first reported case of iatrogenic botulism related to its injection into the abdominal wall musculature. Despite its rarity, the severity of this potential complication underscores the importance of this knowledge for surgeons worldwide.
